# Redox Systems, Oxidative Stress, and Antioxidant Defences in Health and Disease

**DOI:** 10.3390/antiox10121955

**Published:** 2021-12-07

**Authors:** Mario Allegra

**Affiliations:** Department of Biological, Chemical and Pharmaceutical Sciences and Technologies, Università degli Studi di Palermo, Via Archirafi, 28, 90123 Palermo, Italy; mario.allegra@unipa.it; Tel.: +39-091-238-96803

Reactive oxygen and nitrogen species (RONS) play a key role in the regulation of cell survival.

While adequate levels of RONS are essential to sustain cell proliferation and survival, disruption of the endocellular redox state induces cell dysfunction and death. Indeed, under physiological conditions, a balance between the generation and elimination of RONS ensures the proper function of redox-sensitive signalling proteins. Conversely, alterations to the redox homeostasis may disrupt the function of key transcription factors, signal-transduction pathways and cell-death regulators. Along these lines, understanding the mechanisms underlying cellular redox homeostasis may help to develop nutraceutical and/or pharmacological tools to counteract the development of a wide number of redox-dependent pathologies, including cardiovascular, neurodegenerative, inflammatory-based diseases and cancer.

The special issue has brought together updated research concerning molecular mechanisms underlying the control of redox-regulated cell systems in physiological processes and pathological conditions. In addition, recent evidence on the role of phytochemicals, nutraceuticals and dietary patterns in the control of redox-dependent pathophysiological conditions has also been included. New information has been added to this field by means of 15 articles, with 11 original papers and 4 reviews.

In the first paper, Lin et al. have mechanistically evaluated the effects of Gossypetin (GTIN) on abnormal vascular smooth muscle cells’ (VSMCs) proliferation and migration, a major event in the pathogenesis of atherosclerosis. Interestingly, reported results demonstrate that GTIN, at non cytotoxic concentrations, abolish VSMCs’ proliferations. A downregulation of Akt/NF-κB-mediated MMP-9 production and an upregulation of the p53-mediated cell-cycle arrest emerge as key mechanisms of the in vitro effects of GTIN on abnormal VSMCs’ proliferation and migration. These findings suggest that the protective effects of GTIN on VSMCs’ dysfunction might well be a relevant mechanism to explain the previously reported anti-atherosclerotic effects of GTIN [1]. 

Natural antioxidants have repeatedly been reported to counteract the UV radiation-induced alteration of human skin cells. Along these lines, the work by Gęgotek et al. has analysed the protective effects of the hydrophilic ascorbic acid and of the partially lipophilic rutin against UVA/UVB-induced damage in both keratinocytes and fibroblasts. Relevantly, authors demonstrated that, despite the stronger antioxidant properties of ascorbic acid, the lipid membranes were more effectively protected against UV-induced oxidation by rutin in terms of changes in phospholipid fatty acid levels, inhibition of reactive aldehydes’ formation and endocannabinoids’ degradation. On the other side, ascorbic acid more efficaciously prevented UV-induced endocannabinoid receptors’ expression in fibroblasts. As expected, the combination of both antioxidants resulted in the greatest cytoprotective and anti-inflammatory effects. Authors suggested that combined antioxidant capacities of ascorbic acid and rutin protects against lipid peroxidation but also decreases the UV-induced inflammation by direct interaction with the endocannabinoid system, thus increasing skin cell viability [2].

The importance to evaluate natural products’ toxicity on normal cells is a key issue to anticipate their limits and benefits as potential anticancer drugs. In the light of the scanty studies on the cytotoxicity of Usnea barbata (U. barbata) extracts and usnic acid (UA) on normal cells, Popovici et al. have evaluated their cytotoxic potential on human blood cells. Interestingly, authors demonstrated a concentration-dependent cytotoxic effect for all the extracts, with UA inducing the most significant response. At higher concentrations, UA and U. barbata extracts induced apoptosis and DNA damage in human blood cells, through an ROS-mediated mechanism involving the activation of the apoptotic death program [3].

The liver is a key metabolic organ which is particularly sensitive to environmental factors, including UV radiation. In the light of their redox-modulating and anti-inflammatory properties, natural compounds are the object of intense research as potential therapeutic agents against UV-induced oxidative stress and inflammation. Along these lines, Biernacki et al. have assessed the effects of topical application of phytocannabinoid-cannabidiol (CBD) on the skin of nude rats chronically irradiated with UVA/UVB. The results of the study indicate that CBD reaches the rat liver, where it is then metabolised into decarbonylated cannabidiol, 7-hydroxy-cannabidiol and cannabidiol-glucuronide. Interestingly, CBD exerts significant antioxidative effects by increasing levels of GSH and vitamin A after UVB radiation, and preventing the increase of 4-hydroxynonenal (4-HNE) and 8-iso-prostaglandin-F2α levels in UVA-irradiated rats. Moreover, CBD induces relevant anti-inflammatory effects, upregulating levels of the anti-inflammatory 2-arachidonoylglycerol, 15-deoxy-Δ-12,14-prostaglandin J2 and 15-hydroxyeicosatetraenoic acid and downregulating prostaglandin E2 and leukotriene B4 synthesis. Authors conclude that CBD topically applied prevents ROS- and enzyme-dependent phospholipid metabolism in the liver of UV-irradiated rats, suggesting that it could be employed as novel protector agent to counteract the negative effects of UV radiation [4]. 

Endothelium plays an important role in the regulation of both vascular tone and blood fluidity by balancing the production of endothelium-derived vasodilator and vasoconstrictor factors. In their work, López–Fernández–Sobrino have evaluated the involvement of endothelial-derived factors on the blood pressure (BP)-lowering effect of a wine lees powder (WLPW) from a Cabernet grape variety, focusing on the role of SIRT1. Interestingly, authors demonstrated that the antihypertensive effect of WLPW was related to its high content in flavanols and anthocyanins, mediated by alterations in NO availability and reduced by sirtinol, indicating that WLPW decreased BP in a SIRT1-dependent manner. Furthermore, WLPW upregulated eNos and Sirt1 and downregulated Nox4 and Et1 endothelial gene expression. As a whole, these results provide evidence of the vasoprotective effects of WLPW, and show how its antihypertensive effect is endothelium-dependent and mediated by SIRT1 [5].

In their work Cordaro et al. have assessed the effects of Hidrox^®^, an aqueous extract of olive pulp containing hydroxytyrosol, on endometriotic lesions associated with pro-oxidative alterations and pain-like behaviours. Interestingly, authors demonstrated that Hidrox^®^ treatment reduced the endometriotic implant area, diameter and volumes. Mechanistic investigations revealed that Hidrox^®^ treatment was able to counteract mast cell recruitment into the lesions, myeloperoxidase activity and lipid peroxidation, while increasing superoxide dismutase activity and glutathione levels in the endometrial explants. Moreover, in the peritoneal fluid, Hidrox^®^ reduced interleukin (IL)-1β, IL-2, IL-6, tumour necrosis factor-α (TNF-α), and vascular endothelial grow factor levels. Relevantly, Hidrox^®^ administration ameliorated peripheral and visceral sensibility, as shown by behavioural tests. Noteworthy, Hidrox^®^ also reduced blood–brain barrier permeability and mast cell infiltration in the hippocampus, as well as astrocyte and microglia activation. Finally, brain oxidative status was improved by the treatment that restored brain-derived neurotrophic factor protein expression and increased nuclear factor erythroid 2-related factor nuclear translocation. Authors concluded that Hidrox^®^ ameliorated endometriotic dysfunctions and related pain-induced behaviours thanks to its relevant antioxidative properties [6].

Taurine chloramine (TauCl) is an endogenous anti-inflammatory molecule derived from taurine. In their work, Kim et al. have investigated the protective effects of TauCl on experimentally induced colon inflammation. Interestingly, authors demonstrated that TauCl oral administration protected against mouse colitis induced by 2,4,6-trinitrobenzene sulfonic acid (TNBS), attenuating apoptosis in the colonic mucosa of TNBS-treated mice. This effect was accompanied by a reduction of 4-HNE, TNF-α, IL-6 and cyclooxygenase-2 levels in mouse colon. TauCl-mediated effects were accompanied by an inhibition of NFκB and STAT3 activation, and by a stimulation of Nrf2 signalling. As a consequence, Nrf2 target genes encoding heme oxygenase-1, NAD(P)H:quinone oxidoreductase, glutamate cysteine ligase catalytic subunit, and glutathione S-transferase were also upregulated. As a whole, these results suggest that TauCl exerts protective effects against colitis through an upregulation of the Nrf2-dependent cytoprotective gene expression, and an inhibition of NFκB- and STAT3-mediated proinflammatory signalling [7].

The effects of sun exposure on the skin and, specifically, those related to pigmentation disorders are well known. It has, indeed, been shown that blue light leads to the induction of oxidative stress and long-lasting pigmentation. Along these lines, the work by Portillo et al. was designed to simulate chronic exposure to blue light emitted by digital devices on HDF and melanocytes in vitro and to evaluate the photoprotection effects of the hydrophilic, botanical extract from Polypodium leucotomos Fernblock^®^. Interestingly, authors demonstrated that pretreatment with Fernblock^®^ prevents cell death, alteration of mitochondrial morphology, and phosphorylation of p38 in HDF exposed to blue light. In addition, Fernblock^®^ significantly reduced the activation of Opsin-3 in melanocytes and the photo-oxidation of melanin, preventing its photodegradation. Authors concluded that Fernblock^®^ exerts beneficial effects against the detrimental impact of blue light from digital devices and could prevent early photoaging, while maintaining skin homeostasis [8].

Cisplatin resistance remains a significant obstacle that limits the improvement of the clinical outcome of ovarian cancer patients. Cisplatin is an important inducer of intracellular ROS, triggering cancer cell death. Sirtuin 2 (SIRT2) has been reported to regulate cancer hallmarks, including drug responses. Along these lines, in their work, Wang et al. aimed to identify the role of SIRT2 in oxidative stress and cisplatin response in cancer cells. Interestingly, authors found a different expression pattern of SIRT2 in cisplatin-sensitive (A2780/S) and cisplatin-resistant (A2780/CP) cancer cells, whereas SIRT2 expression was augmented only in A2780/S cells. Furthermore, cisplatin-induced ROS generation was responsible for the upregulation of SIRT2 in A2780/S cells, whereas overexpression of SIRT2 significantly enhanced the sensitivity of cisplatin-resistant counterpart cells to cisplatin. Authors propose that targeting SIRT2 may provide new strategies to potentiate platinum-based chemotherapy in ovarian cancer patients [9].

Rosmarinic acid (RosA) has been widely investigated for its antioxidant and anti-inflammatory properties. Conversely, less attention has been devoted to its application in theranostics. Along these lines, Kim et al. synthesised a gadolinium (Gd) complex of 1,4,7,10-tetraazacyclododecane-1,4,7-trisacetic acid (DO3A) and RosA (Gd(DO3A-RosA)(H2O)) to evaluate its use as a single-molecule theranostic agent. In their paper, authors demonstrated that its kinetic stability was comparable to that of clinically used, macrocyclic magnetic resonance imaging contrast agents. In addition, its relaxivity was higher than that of the structurally analogous Gd-BT-DO3A. The new agent was evaluated for inflammatory targeting magnetic resonance contrast and showed strong and prolonged enhancement of imaging in inflamed tissues of mice. The theranostic molecule also possessed antioxidant and anti-inflammatory properties, as evidenced by its ability to behave as a ROS scavenger, and to modulate superoxide dismutase, cyclooxygenase-2 and TNF-α expression. The novel RosA-conjugated Gd complex could be envisaged, then, as a promising theranostic agent for the imaging of inflamed tissues, as well as for the treatment of inflammation and oxidative stress [10].

Although some lichens have been widely investigated by traditional medicine and showed interesting anticancer effects, less attention has been paid to U. barbata. Along these lines, Tang et al. have evaluated the antiproliferative effects of U. barbata methanolic extracts (MEUB) against oral cancer cell proliferation. Relevantly, authors demonstrated that MEUB shows a remarkable selectivity vs. several oral cancer cell lines (Ca9-22, OECM-1, CAL 27, HSC3, and SCC9) in comparison to normal oral cell lines (HGF-1). More specifically, Ca9-22 and OECM-1 cells display the highest sensitivity to MEUB, which induced apoptosis, oxidative stress and DNA damage. From a mechanistic perspective, the MEUB-induced antiproliferative effects were mediated by an alteration of endocellular oxidative stress, as demonstrated with parallel experiments with *N*-acetylcysteine [11]. 

In their paper, Gkekas et al. reviewed the effect of polyglutamine (polyQ)-induced oxidative stress in cellular and animal models of polyQ diseases. Furthermore, authors discussed the interplay between oxidative stress, neurodegeneration and neuroinflammation using as an example of a well-known neuroinflammatory disease, i.e., multiple sclerosis. Finally, some of the pharmacological interventions which may delay the onset and progression of polyQ disorders by targeting disease-associated mechanisms were also reported [12].

*N*-acetylcysteine (NAC) is both a widely used drug to treat paracetamol overdose and a mucolytic compound. It has a well-established safety profile and remarkable antioxidant and anti-inflammatory properties. The primary role of NAC as an antioxidant stems from its ability to increase the intracellular concentration of glutathione, the most crucial biothiol responsible for cellular redox homeostasis. As an anti-inflammatory compound, NAC can reduce levels of TNF-α, IL-6 and IL-1β by suppressing NF-κB activity. Despite NAC’s relevant therapeutic potential having been evaluated in several experimental studies, its effectiveness in clinical trials, addressing different pathological conditions, is still limited. Along these lines, dos Santos Tenorio et al. provided an overview of the medicinal effects and applications of NAC in human health [13].

Serum albumin is the most abundant circulating protein in mammals. It has three isoforms according to the redox state of the free cysteine residue at position 34, named as mercaptalbumin (reduced albumin), and non-mercaptalbumin-1 and -2 (oxidised albumin), respectively. The serum albumin redox state has long been viewed as a biomarker of systemic oxidative stress, as it shifts to a more oxidised state in response to the severity of pathological conditions such as liver diseases and renal failures. Moreover, recent ex vivo studies revealed that oxidised albumin *per se* could aggravate these pathological conditions. Furthermore, the role of the serum albumin redox state as a nutrition-related, sensitive, protein biomarker has also been demonstrated in a series of animal studies. The work from Tabata et al. provides an updated overview on the analytical techniques to evaluate the serum albumin redox state and its association with human health [14].

Like other post-translational modifications of proteins, S-nitrosylation has been regarded a key, regulatory mechanism of multiple cellular functions in many physiological and pathological conditions. Indeed, a wealth of evidence has demonstrated that S-nitrosylation plays a crucial role in regulating redox homeostasis in the stressed heart. This has led to a thorough evaluation of the mechanisms underlying the pathogenesis of heart diseases and cardiac protection. In their paper, Shi et al. reviewed recent studies devoted to understanding the molecular and biological basis of S-nitrosylation, including the formation, spatiotemporal specificity, homeostatic regulation and association with cellular redox status. The currently available methods that have been applied to detect S-nitrosylation have also been reviewed together, with up-to-date therapeutical studies of S-nitrosylation in various in vivo models of cardiac diseases [15].
